# Optic perineuritis secondary to hyaluronic acid injections: a case report

**DOI:** 10.1186/s12886-019-1247-2

**Published:** 2019-11-27

**Authors:** Yanjun Hu, Yingjun Wang, Yuhua Tong

**Affiliations:** People’s Hospital of Quzhou, 2 Zhongloudi Road, Quzhou, Zhejiang 324000 People’s Republic of China

**Keywords:** Hyaluronic acid, Complication, Eyebrow, Optic perineuritis

## Abstract

**Background:**

Although a safe, excellent administration method for hyaluronic acid derivatives has been documented; improper injections can lead to devastating and irreversible consequences. Here, we present the first known case of optic perineuritis caused by hyaluronic acid.

**Case presentation:**

A young female experienced sudden orbital pain in the right eye after receiving hyaluronic acid injections to the eyebrows. She presented to the eye clinic two weeks later, after developing blurred vision in the right eye. Visual acuity was reduced significantly in the right eye. Automated visual field examination showed defects in both eyes. Fundus examination revealed bilateral swelling of optic discs. Magnetic resonance imaging of the brain demonstrated bilateral perineural enhancement consistent with optic perineuritis. The patient was treated with retrobulbar injection of hyaluronidase and oral prednisolone. Her vision improved with treatment.

**Conclusions:**

The prognosis for visual outcomes in patients with optic perineuritis is generally excellent. However, a poor prognosis is associated with delays to the initiation of treatment. Recognizing this condition is important, and treatment with corticosteroids should be initiated early.

## Background

The use of soft-tissue fillers for cosmetic purposes has increased dramatically in recent years. Hyaluronic acid (HA) is a naturally occurring linear polysaccharide found in the extracellular matrix of connective tissue, synovial fluid, and other tissues. HA was first produced approximately 80 years ago and was approved by the FDA as a dermal filler for the correction of wrinkles; it remains the most popular filler [1, 2].

Here, we report a case of optic perineuritis (OPN) after HA filler injection into the eyebrow. OPN is a rare disorder which is clinically indistinguishable from retrobulbar optic neuritis. The prognosis, treatment, and follow-up care are quite different for these two entities [3]. In this case report, an unusual adverse event, OPN, was observed after the injection of HA; in addition, medical records and imaging were reviewed to better characterize the clinical features of OPN.

## Case presentation

A 22-year-old female patient underwent the cosmetic injection of HA for her eyebrows in an illegally operated clinic. HA (1.1 ml) was injected under each eyebrow. A few seconds after the injection needle was withdrawn, the young woman suffered orbital pain on the right side of the eye. Hyaluronidase (100 U) was injected under the right eyebrow immediately to degrade the HA. After 3 days, rotation pain occurred in both eyeballs, and the patient was sent to the ophthalmology department. A physical examination demonstrated that the pupillary light reflex was normal; additionally, fundus imaging and an orbital computer tomography scan were normal. However, no electrophysiological examinations were performed, and the pain spontaneously relieved but later recurred.

**Fig. 1:**
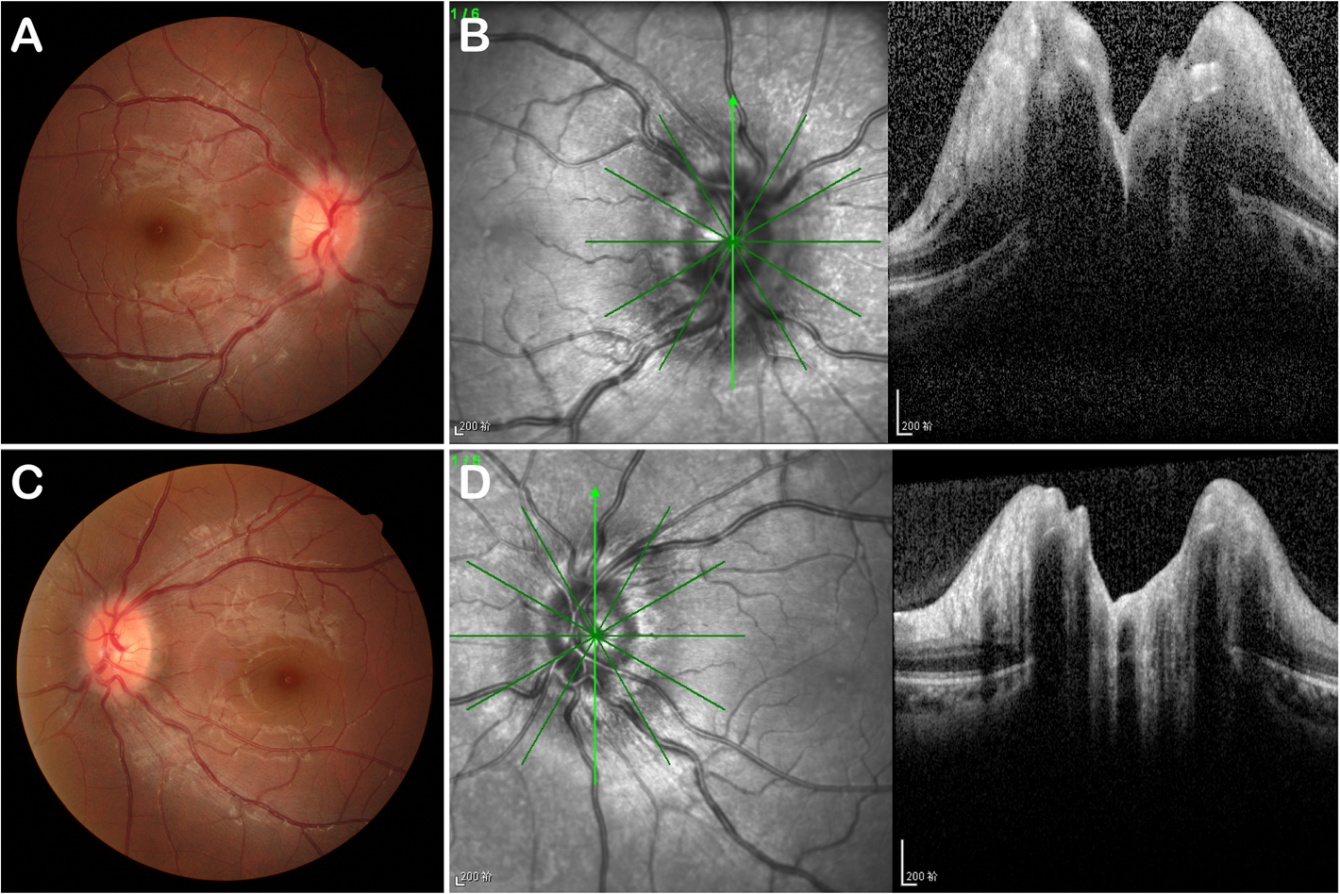
Fundus photograph and ocular coherence tomography. Fundus photograph (**a** and **c**) and ocular coherence tomography (**b** and **d**) obtained at the initial visit showing papilloedema, tortuous venous twisting and dilation. **a** and **b** The right eye; **c** and **d** the left eye

**Fig. 2:**
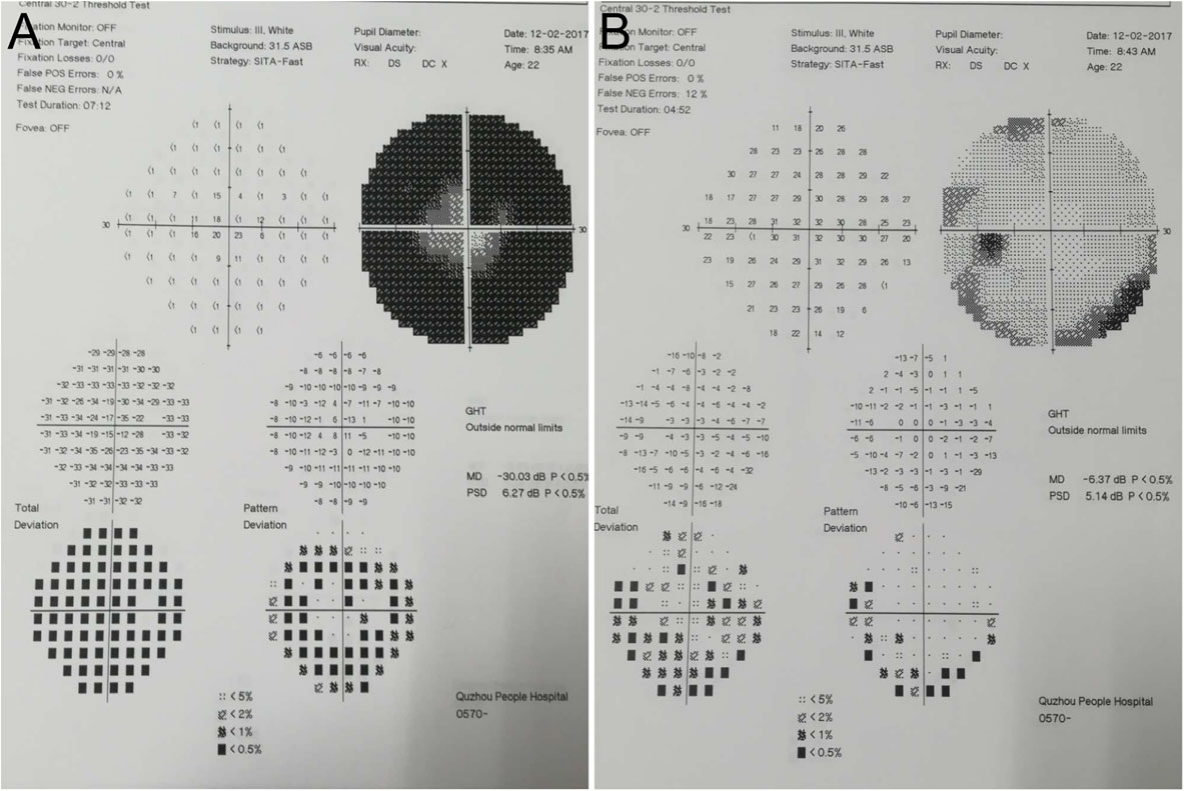
Visual field. **a** The right eye showed a tubular visual field. **b** Peripheral vision was narrow in the left eye

**Fig. 3:**
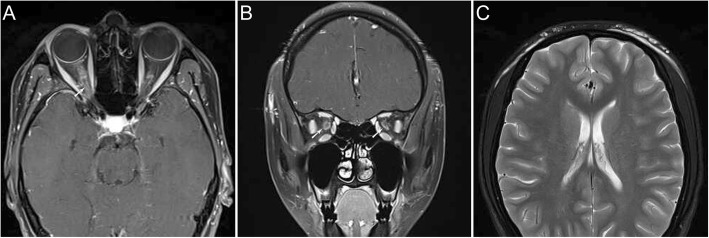
Magnetic resonance imaging. Fat-saturated T1-weighted MRI of this patient. In the axial plane (**a**), a short length of the optic nerve sheath showed tram-track enhancement (arrowed). (**c**), the lateral subcutaneous fat layer on both sides of the forehead had a band-like abnormal signal that was more pronounced on the left side. T1 WI had an equal signal, T2 WI had a high signal, the boundary was clear, and the medium intensity uneven enhancement was enhanced. In the coronal plane (**b**) circumferential optic nerve sheath enhancement is demonstrated (arrowed)

Two weeks later, she had blurry vision in her right eye; thus, she came to the department of ophthalmology. No oedema or lesion was found around the injection point. The best-corrected visual acuity at initial presentation was 20/32 in the right eye and 20/20 in the left eye. Fundus imaging of both eyes showed papilloedema and venous tortuosity and dilation, especially in the right eye (Fig. 1). Computer automated visual field examination of the right eye showed tunnel vision, while the left eye exhibited peripheral depression (Fig. 2). A magnetic resonance imaging (MRI) scan of the head and orbits showed bilateral optic nerve sheath thickening. No obvious oedema of the extraocular muscles was observed. The lateral subcutaneous fat layer on both sides of the forehead had a band-like abnormal signal that was more pronounced on the left side. T1 WI had an equal signal, T2 WI had a high signal, the boundary was clear, and the medium intensity uneven enhancement was enhanced (Fig. 3). Extraocular movements and anterior segment examination including intraocular pressure were normal in both eyes. The relative afferent pupillary defect test was performed. Laboratory testing included the determination of the complete blood cell count, the thyroid function test, rheumatoid factor, anti-streptococcus haemolysin O antibody, erythrocyte sedimentation rate, levels of antinuclear antibodies and antineutrophil cytoplasmic antibodies, syphilis serologic test and chest radiography. The results of all tests were normal or negative. She had no history of prior ocular or systemic disease and no allergies to medications or known substances.

The patient was diagnosed with OPN secondary to HA. Hyaluronidase (150 U) was immediately injected into the retrobulbar region of each eye. Oral prednisone treatment was started at 80 mg/d and decreased by 20 mg/wk. After 2 days of treatment, visual acuity was 20/20 in both eyes. At the 3-day follow-up, the visual field had improved, and the fundus examination revealed decreased oedema of the optic disc. During the treatment, the patient did not complain of insomnia, stomachache or any other discomfort.

## Discussion

Adverse events can occur with improper soft-tissue filler usage. Mild events include ecchymosis, swelling, and erythema. Severe events, such as visual impairment, skin necrosis, and anaphylaxis, have been reported [4–9]. Ophthalmic artery occlusion is the most devastating complication, and although it is rare but has been reported many times [10–13].

This case is unique in that it represents the first known case of OPN caused by HA.

First described in 1883, OPN encompasses a range of disorders characterized by pathologic inflammation of the optic nerve sheath, usually presenting with the classic triad of pain, optic neuropathy, and optic disc swelling [14]. Visual acuity is varied, and associated visual field abnormalities include arcuate defects, paracentral scotomas, central scotomas, and peripheral island defects. The diagnosis of OPN itself can be confirmed by the histopathologic or radiographic demonstration of peri-neural inflammation. The typical appearance on MRI is circumferential optic nerve sheath enhancement. Once OPN has been diagnosed, treatment with corticosteroids should be started at a high dose if vision is severely affected [3, 15–18].

Our patient’s symptoms, signs, fundus imaging, MRI results and response to corticosteroids were suitable for a diagnosis of OPN. There was no significant positive laboratory serum test. The patient was not allergic to medications or known substances and had no history of prior ocular or systemic disease. No systemic disease was identified except for the HA filler. Thus, HA was likely the cause of OPN.

With the direct puncture of vessels, HA may obstruct blood flow, resulting in symptoms of ischaemia in the eye or local tissue [7, 8]. Unfortunately, fundus fluorescein angiography was not performed on the patient to rule out the possibility of vascular blockage. The use of HA is associated with a potential inflammatory response [19]. HA may induce oedema, haemorrhage, or inflammation. Alternatively, histological and clinical examinations have documented intermittent swelling and severe granulomatous allergic reactions associated with the use of HA [20]. Fillers can enhance eyebrow contour and volume and may be used for improving the elevation of the eyebrow tail. A histologic examination revealed the location of the hyaluronic acid gel, and the dense retro-orbicularis oculi fat septa appeared to limit the displacement of the filler [21]. The orbital rim and supraorbital foramen should be carefully identified to avoid inadvertent injection into the orbital cavity [22]. As our patient has an Asian background, the orbital septum joins the levator below the tarsus, resulting in migration from the brow down into the fat pads and retro-orbital space, accounting for the observed oedema and inflammatory reaction [23]. This issue could be the cause of secondary OPN in this case. However, we still found one issue confusing. HA was injected under each eyebrow simultaneously, and hyaluronidase was injected immediately after eye pain occurred in the right eye. Why the pain, blurry vision, optic disc oedema and visual field damage were more severe on the right side has remained unclear. Hyaluronidase is used off-label to dissolve HA and reverse and manage situations such as excessive use of HA, very superficial injections, a granulomatous foreign-body reaction, and injection necrosis [24]. Therefore, we wonder whether a deviation in the injection site resulted in the differences between effects on each side [20]. The injection of hyaluronidase is performed solely to reduce the risk of arterial obstruction. It has been well established that corticosteroids prednisolone is the treatment of choice in optic perineuritis.

In summary, we present the first known case of OPN secondary to HA. HA filler injection should be carefully applied, especially in the periorbital area, by an experienced physician with a good understanding of the anatomy of the facial vasculature. OPN is a rare disorder that is clinically indistinguishable from retrobulbar optic neuritis. A poor prognosis is associated with a delay to the initiation of treatment; thus, clarifying the diagnosis of OPN and prompt treatment with corticosteroids should be performed.

## Data Availability

Not applicable.

## Abbreviations

HA: Hyaluronic acid
MRI: Magnetic resonance imaging
OPN: Optic perineuritis
